# Fluorescent Chemosensors in the Creation of a Commercially Available Continuous Glucose Monitor

**DOI:** 10.1021/acssensors.4c02403

**Published:** 2024-11-25

**Authors:** Anthony W. Czarnik, Tony D. James

**Affiliations:** † Department of Chemistry, University of Nevada, Reno, Reno, Nevada 89511, United States; ‡ Department of Chemistry, University of Bath, Blaverton Down, Bath BA2 7AY, United Kingdom

**Keywords:** fluorescent chemosensor, diabetes, glucose, boronic acids, continuous monitoring, artificial pancreas

## Abstract

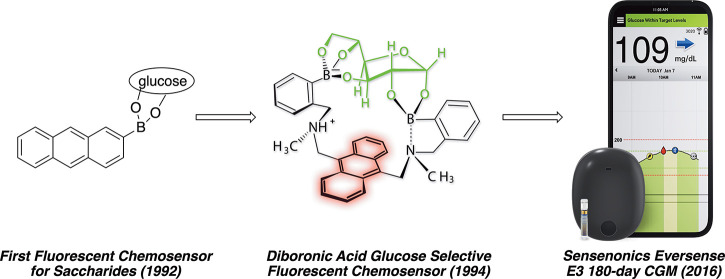

Fully automated insulin delivery (i.e., an artificial pancreas) would revolutionize diabetes disease management, minimize negative secondary disease outcomes, and simultaneously reduce health care costs and system burdens. Continuous glucose monitoring (CGM) is an essential aspect of the artificial pancreas. Abiotic fluorescent chemosensors play a key role in generating long-lived CGM sensors for this purpose. In this Perspective, we detail our initial discoveries of chemosensors for saccharides, as well as the development and advancement of bis((*o*-aminomethylphenyl)boronic acid)anthracene-based sensors for commercial use. While a few companies have sought to bring a copolymerized diboronic acid CGM sensor to the market, Senseonics is the only one, to date, to have done so. In this case, the system has been approved in the U.S. and Europe to provide accurate CGM for up to 365 days with a single sensor and can be integrated directly with an insulin pump, bringing an artificial pancreas one step closer to realization.

The ability to monitor biological processes in real time has dramatically changed the modern health care landscape. Today, simple fitness trackers and smart watches monitor an array of health markers (e.g., heart rate, respiration, pulse oxygen levels, and body temperature), allowing millions of people and their health care providers to detect adverse health events and illness earlier than ever before.

The plethora of emerging commercial devices continues to expand the types of real-time monitoring that is possible (e.g., glucose monitoring, heart function, etc.).^1^ In 2024, the Center for Disease Control’s National Diabetes Statistics Report found that, in 2021, the most recent year for which data are available, 38.4 million Americans (11.6%) were afflicted with diabetes, while an additional 97.6 million citizens (38.0% of the U.S. population) are considered to be prediabetic.^2^ With nearly half of the U.S. population either suffering from or at risk of developing diabetes, devices that manage blood glucose levels are critical to reducing disease burden on individuals as well as managing health related costs to society.^3,4^

For those suffering from insulin-sensitive diabetes, technologies for continuous glucose monitoring have been shown to significantly improve disease management (e.g., increased time in the target glucose range, decreased hemoglobin A1c (HbA1c) levels, which indicate how well controlled an individual’s glucose levels have been over the past 2–3 months),^5^ relative to patients who rely on self-monitoring blood glucose (SMBG) levels. Continuous glucose monitors (CGMs) utilize a sensor that complexes to glucose, which is either inserted through the skin by the patient or fully implanted under the skin by a health care professional. Upon complexation to glucose, a signal is transmitted to a receiving device that correlates the signal to the patient’s blood glucose level. Currently, short-term (i.e., 14 days or less) enzyme-based *biosensors*, such as those available from companies such as Medtronic Diabetes Care, DexCom, and Abbott, are utilized by the majority of CGM users; however, the short lifetimes of these biosensors require patients to insert a new device through the skin every 7–14 days.^6^ By comparison, abiotic *chemosensor*-based CGMs (e.g., Senseonics Eversense) are implanted by a health care professional and can provide accurate glucose measurements for a year without needing to be changed.^7^

## Origins of the Chemosensor Field

A chemosensor is a molecule of abiotic origin that signals the presence of matter or energy.^8^ Compounds that incorporate a binding site, a fluorophore, and a means for communicating between the two are known as *fluorescent chemosensors*.^8^ Fluorescent chemosensors are particularly useful for biological real-time applications, such as CGM, given that fluorescence can be detected through biological tissue.^9^

The first reports of naturally occurring molecules fluorescing in the presence of metal ions was reported in the mid-19th century.^10,11^ These early studies utilized compounds such as morin (2′,3′,4′,5,7-pentahydroxyflavone) to generate highly fluorescent complexes with metal ions. The abundance of electronegative heteroatoms in such compounds likely explains why metal ions, rather than anions or neutral compounds, were first detected from aqueous environments.

Yet, the ability to detect neutral species from water, such as saccharides, represents a significant opportunity to positively impact human health. In fact, the very first article published in the *Journal of the American Chemical Society* (1879) reported the measurement of glucose concentrations.^12^ As discussed above, determining blood glucose levels in a continuous fashion offers the ever-growing diabetes patient community a new option for disease control and management. With this goal in mind, many laboratories began to design abiotic fluorosensors in the 1970s.^13^ One class of sensors, *conjugate chemosensors*, utilizes a heteroatom-based binding domain that is insulated from a fluorophore π-system. In this way, binding and fluorescence are integrated without restricting the steric environment of either.^11^ Advances in this area have extended fluorescent chemosensing to a vast range of applications, including bio-imaging, detection of environmental pollutants, phosphates, transition metals, lanthanides, and reactive oxygen, sulfur, and selenium species, determining chirality, and targeted cancer drug delivery.^14,15^

## Glucose Sensing

**Figure 1:**
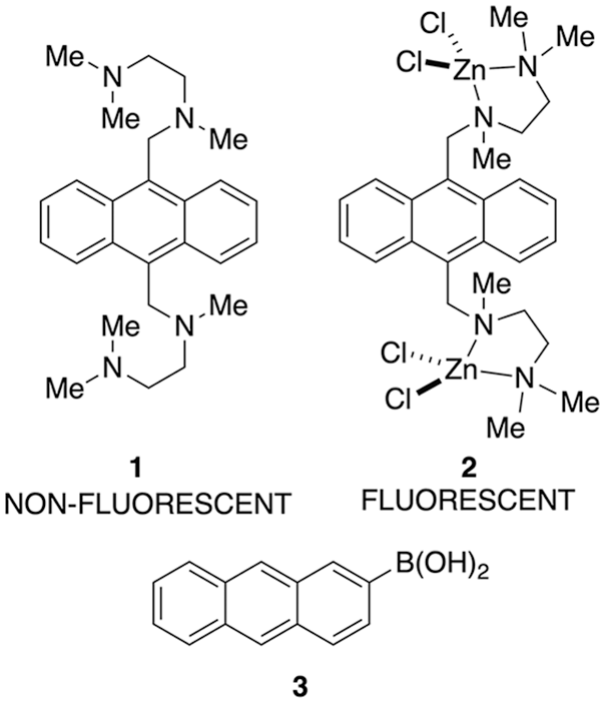
Initial anthracene-based fluorescent chemosensors.

Early work from Czarnik’s research group at the Ohio State University showed that 9,10-bis[[2-(dimethylamino)ethyl]methylamino]methyl]anthracene (**1**), which is nonfluorescent, became highly fluorescent upon complexation to ZnCl_2_ (**2**, Figure 1).^16^ Encouraged by Moore and co-workers’ observation that boronic acid impregnated cellulose columns were able to separate ribonucleotides from deoxyribonucleotides through the formation of transient boronic esters,^17^ we incorporated a boronic acid into anthracene **3**. When exposed to glucose or fructose, anthracene **3** became fluorescent; the first abiotic fluorescent chemosensor for aqueous solutions of monosaccharides had been discovered!^18^ In this case, formation of transient boronic ester linkages between the alcohols of the saccharide and the boronic acid outcompete hydrogen bonding—even in aqueous solution—overcoming a significant barrier to sensing physiological glucose.

**Figure 2:**
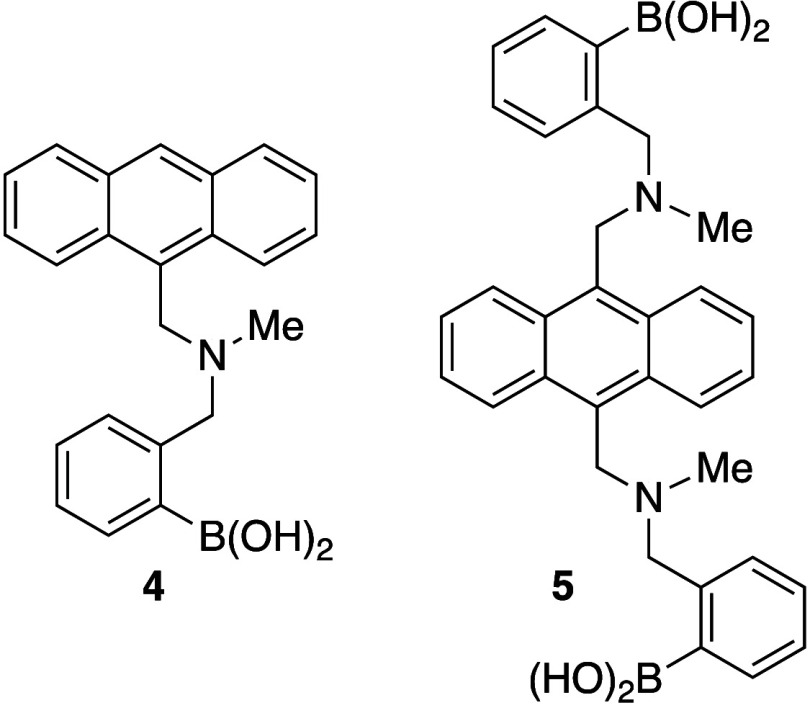
Enhanced fluorescence sensors for saccharides.

**Figure 3:**
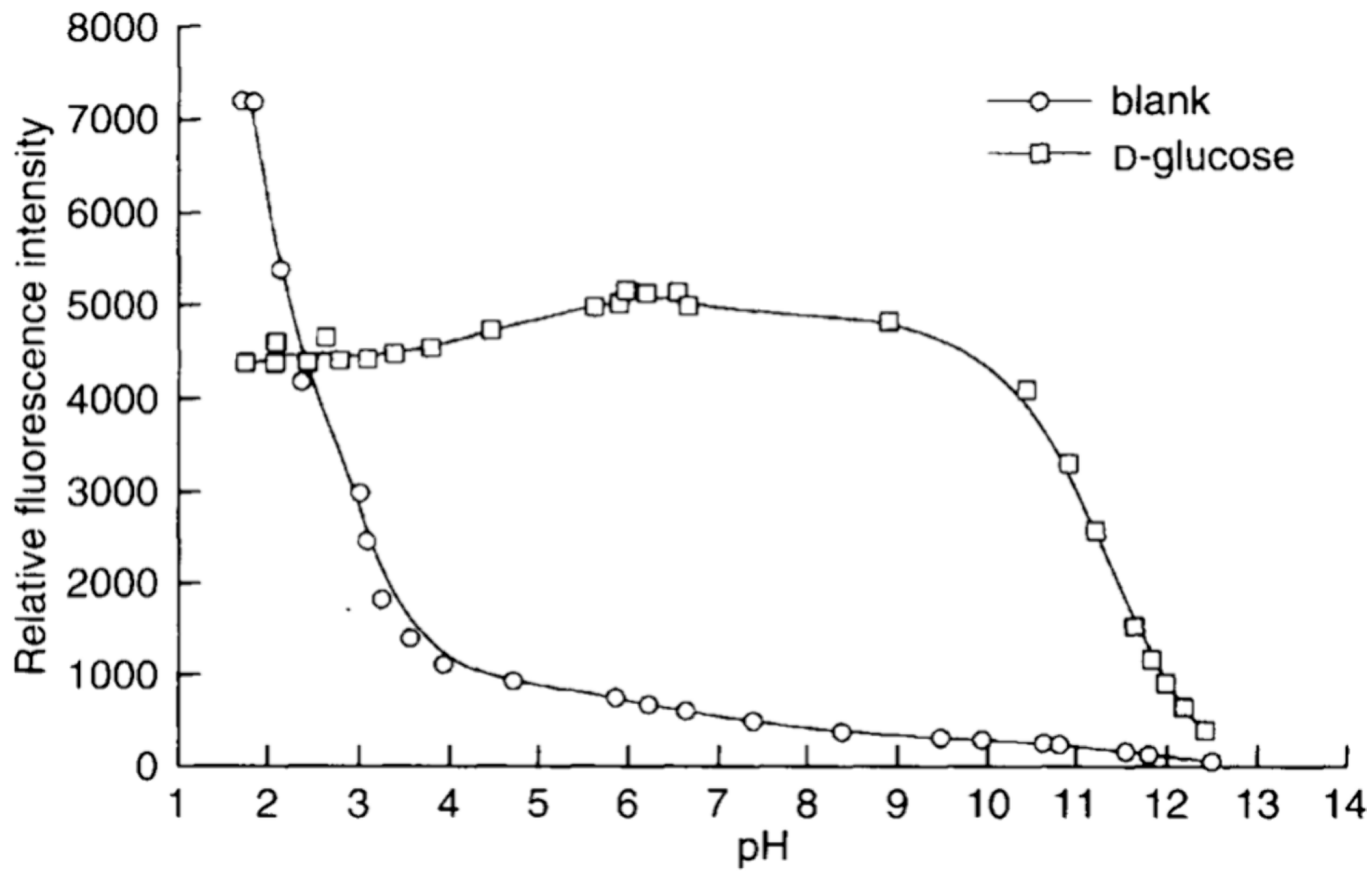
Fluorescence intensity vs pH profile of compound **4** at 25 °C and 1.2 × 10^–5^ mol dm^–3^**7** in 0.05 mol dm^–3^ sodium chloride solution. [glucose] = 0.05 mol dm^–3^. Reproduced with permission from ref (22). Copyright 1996 Royal Society of Chemistry.

This proof of concept quickly ignited interest in boronic acid based fluorescent chemosensors for saccharides.^19^ Of particular note were sensors with selectively for glucose over other physiologically important saccharides (e.g., galactose, fructose)—a selectivity that was absent in anthracene **3**. As a postdoctoral fellow in the lab of Shinkai, James found that linking an (*o*-aminomethylphenyl)boronic acid to the anthracene, as in sensor **4**, amplified the change in fluorescence between free sensor **4** and the saccharide bound complex, relative to that observed for anthracene **3** (Figure 2).^20^ Further, the enhancement in fluorescence was present over a large pH range in aqueous environments, allowing sensor **4** to function at physiological pH (Figure 3). Incorporating a second boronic acid, as in analogue **5**, provided the first fluorescent chemosensor that was selective for glucose over other monosaccharides; sensor **5** maintains its enhanced fluorescence over a broad pH range.^21^

**Figure 4:**
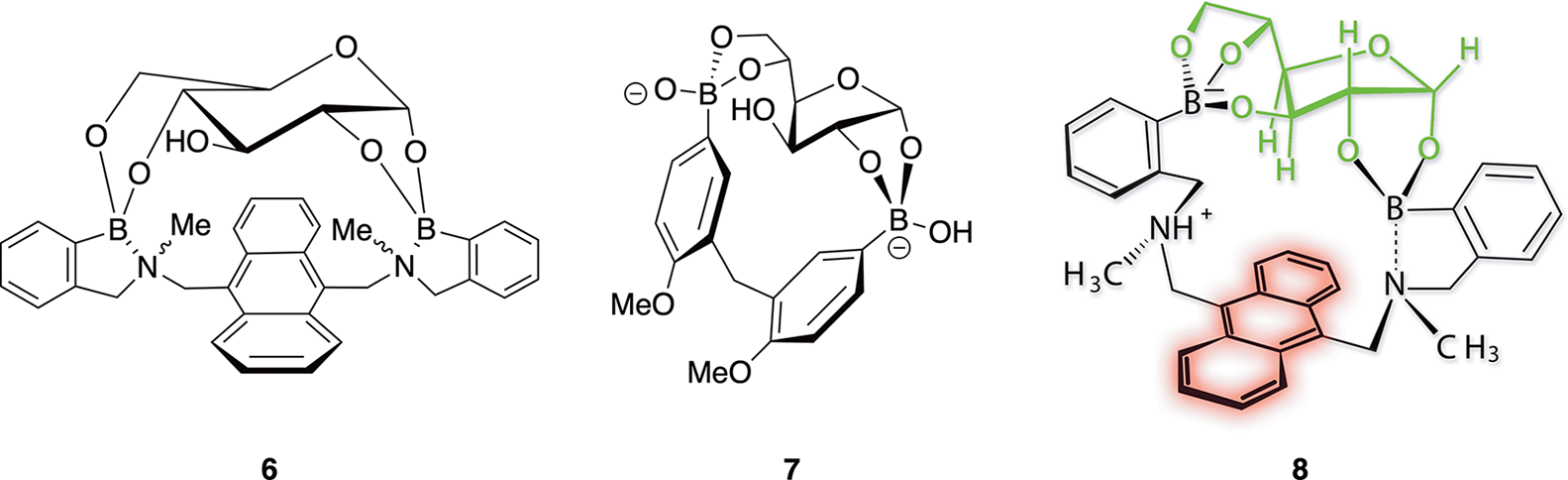
Structures of sensors bound to pyranose and furanose isomers of glucose.

Initial NMR and mass spectrometry data of glucose bound diboronic acid **5** indicated the formation of a 1:1 complex between glucose and sensor **5** to form complex **6**, whose cleft appears to be ideally sized for glucose (Figure 4).^21^ Moreover, the NMR spectrum suggested that initial glucose binding occurred in the common pyranose form (see complex **6**). Later studies by Eggert showed that in the presence of water and a model sensor that mimics diboronic acid **5**, glucose rapidly isomerizes to its furanose isomer, forming complex **7**.^23^ In its furanose form, five glucose oxygen atoms are engaged in boronic ester bonds. Eggert then showed that glucose also isomerizes to its furanose form in the presence of water and sensor **5** to generate structure **8**.^24^

In the hopes of further increasing the selectivity for glucose, enhancing the fluorescent signal upon binding, and improving water solubility, a range of diboronic acid glucose sensors were synthesized and evaluated by James and co-workers, now working independently at the University of Bath.^25^ Initial studies focused on the linker between boronic acid moieties, evaluating analogues in which the fluorophore was either within the linker or attached as a substituent on one of the aminomethyl groups. When the fluorophore was a substituent, it was found that six-carbon alkane linkers provided the highest levels of glucose selectivity.^26^ Additional studies evaluated the possibility of rigidifying the linker by including aromatic groups; however, in general, six-carbon alkyl linkers were found to be the most promising.^25^

The change in observable fluorescence upon binding glucose make sensors such as (*o*-aminomethylphenyl)boronic acid **5** of interest for CGM. Yet, the mechanism responsible for “turning on” fluorescence upon glucose binding was debated for many decades.^27^ Original reports agreed that the tertiary amine served to quench the inherent fluorescence of the fluorophore. Saccharide binding then disrupts this interaction, leading to the observed fluorescence. The exact nature of the nitrogen–boron interaction, both before and after saccharide binding, and the means of electron transfer that led to fluorescence, however, remained unclear until the late 2010s. Competing mechanisms postulated that either (1) a photoinduced electron transfer (PeT)^28^ occurred upon saccharide binding that increased the bond strength of the N–B interaction (referred to as the N–B bonding mechanism);^20,21^ (2) saccharide binding caused the existing N–B bond to break with concurrent solvent insertion;^29,30^ or (3) aggregation and disaggregation of the sensor fluorophores resulted in the observed changes in fluorescence.^31^ However, none of these mechanisms were entirely consistent with the experimental data.

**Figure 5:**
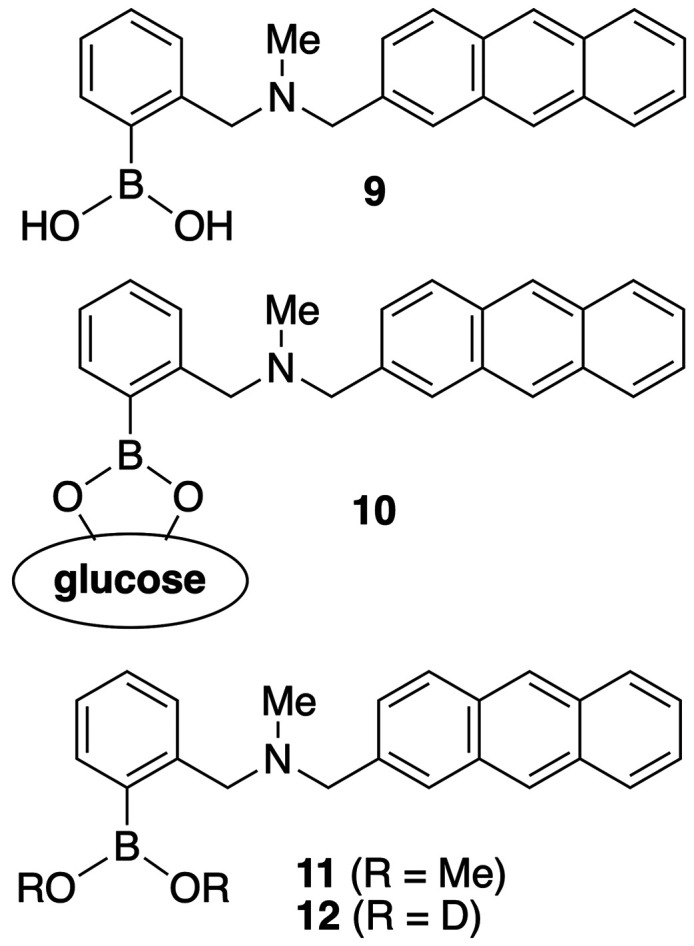
“Loose bolt” mechanism of fluorescence in boronic acid based sensors.

In the hopes of finally determining how saccharide binding switches fluorescence “on”, a collaboration between the laboratories of James and Anslyn began.^32^ Model sensor **9** exhibits no fluorescence in aqueous solution; however, upon saccharide binding, fluorescent boronic ester **10** is formed (Figure 5). Conversely, dimethoxy boronic ester **11** is generated upon exposure of sensor **9** to methanol. In this case, subsequent addition of saccharide results in no change in the fluorescence. Further, if sensor **9** was stirred in D_2_O, rather than H_2_O, there was also no change in the observed fluorescence upon addition of saccharide. These observations suggested that the −OH groups of boronic acid **9** are directly involved in fluorescent quenching and quenching is not possible when the hydroxyl groups are substituted with a heavier substituent (e.g., deuterium, methyl, or saccharide). In particular, we advocate for a “loose bolt” mechanism.^32^ This theory proposes that the vibrational motion of –OH groups, similar to a loose bolt in an engine, allows for the dispersion of energy, leading to boronic acid **9** being nonfluorescent. Upon replacing the hydrogens with heavier groups (e.g., deuterium, methyl, or saccharide), these vibrations are minimized, hindering energy dispersion (i.e., quenching). Thus, compounds **11** and **12**, which cannot disperse energy through protio hydroxyl groups, are found to be fluorescent in the absence of saccharide and exhibit no significant change in fluorescence upon saccharide binding.

## Commercialization Attempts

A number of companies have sought to commercialize polymer bound bis((*o*-aminomethylphenyl)boronic acid)anthracene based sensors for long-term CGM. In 1997, Sensors for Medicine and Science, Inc. (SMSI) was founded with the goal of commercializing fluorescent chemosensor based probes for real-time biological monitoring. In particular, SMSI was interested in advancing a CGM that would reliably measure patient glucose levels in interstitial fluid (ISF) with a lifetime of months to years, in contrast to the 7–14 day lifetimes found in the more commonly used enzyme-based CGM sensors from Medtronic Diabetes Care, DexCom, and Abbott.^33^ In 2001, Czarnik was recruited to serve as the Chief Scientific Officer (CSO) at SMSI, given his early contributions to the boronic acid saccharide sensing field^18^ and his significant entrepreneurial expertise. During his two years as CSO, Czarnik oversaw SMSI’s initial animal studies of bis((*o*-aminomethylphenyl)boronic acid)anthracene based glucose sensors in rabbits. In those studies, a prototype sensor hydrogel was implanted under the skin of a rabbit’s back, and fluorescence was observed through the skin in response to glucose concentrations. These studies provided the first evidence that a hydrogel-based sensor could be used to detect changing glucose levels *in vivo*.

With a similar goal, GlySure Ltd. was launched in the United Kingdom in 2006 with James as a key collaborator. Again, bis((*o*-aminomethylphenyl)boronic acid)anthracene based sensors were targeted for CGM; however, in this case, GlySure initially focused on CGM of blood rather than ISF, leading to an intravascular CGM solution that was shown to be effective for critically ill patients in intensive care units (ICUs).^34^ Application of CGM in the ICU is of great value since changes in blood glucose levels can signal other developing issues within this patient population. Unfortunately, despite the success of the GlySure intravascular system, evolution of a wireless mobile system for CGM of diabetes patients was never achieved.

At the time of this writing, neither author has any current association with SMSI (now Senseonics) or GlySure and neither benefits financially from the sale of any CGM system.

**Scheme 1:**
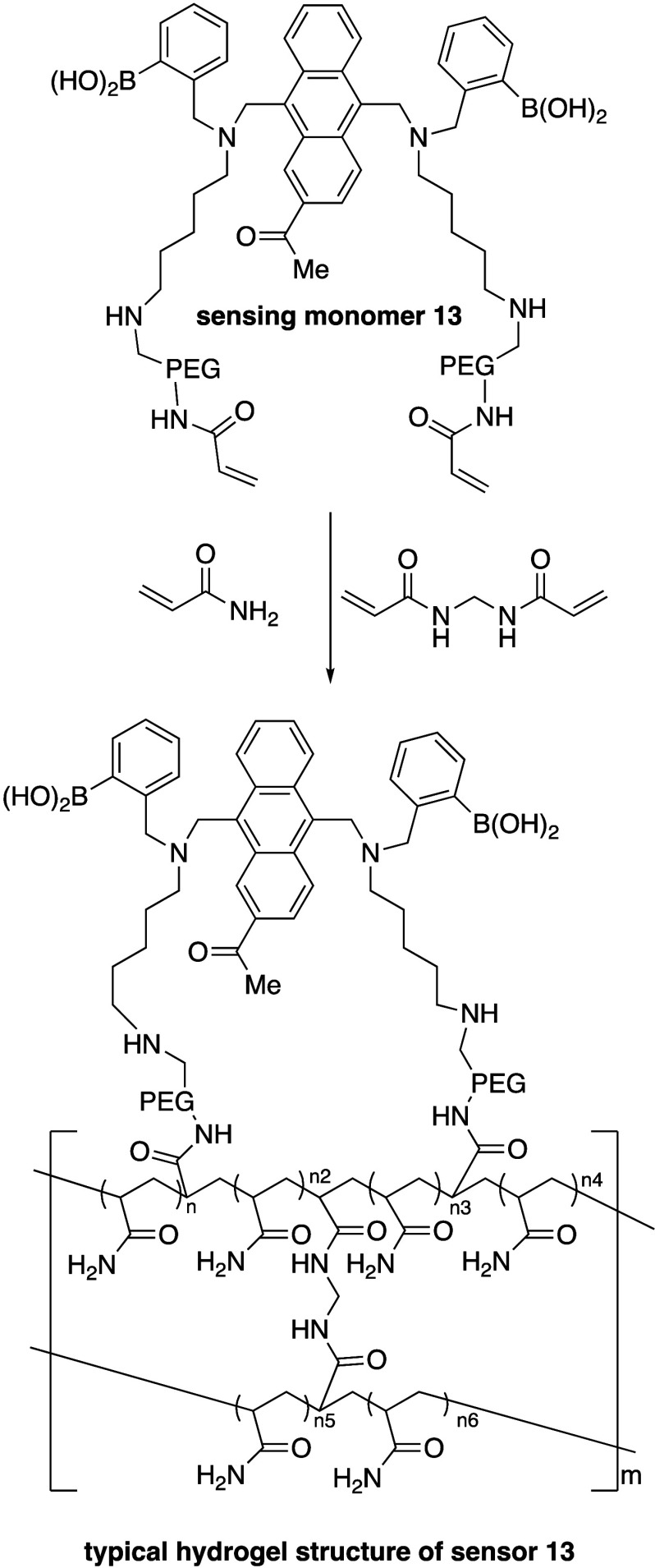
Co-polymer of Polymethymethacrylate and Diboronic Acid Sensor **13**

Despite their early success, many recognized that existing and emerging polymer and nanomaterial technologies would be critical to creating a fully functional sensor that could resist biological fouling and maintain accuracy throughout the extended lifetime of the device. Porous copolymer materials that contained fluorescent chemosensors seemed promising. In 2007, microporated polyethylene glycol (PEG) spheres were examined for this type of fluorescence application.^35^ Subsequently, Takeuchi and co-workers, working together with Shinkai and Terumo Corp.,^36^ reported that injectable hydrogel beads, composed of a poly(methyl methacrylate) (PMMA) and diboronic acid monomer (**13**) copolymer, could be injected into the ear skin of mice and the changes in fluorescence could be readily detected and correlated to known glucose concentrations (Scheme 1).^37^ While the beads were well tolerated and could effectively indicate glucose concentration in ISF, they tended to migrate away from the site of injection, making them difficult to remove from the animals. To overcome this obstacle, hydrogel fibers of a similar composition were investigated. Just like the beads, hydrogel fibers correlated fluorescence intensity with glucose concentration;^38^ however, the fibers were easily removed from the animals at the end of the study. While Terumo Corp. continues to be interested in diabetes technology (e.g., they marketed various DexCom CGM systems in Japan from 2019 to 2024 and developed and commercialized a low profile pen needle for SMBG measurements),^39,40^ advancement of a long-term CGM sensor based on bis((*o*-aminomethylphenyl)boronic acid)anthracene has not emerged within their offerings.

The array of commercial ventures aimed at using bis((*o*-aminomethylphenyl)boronic acid)anthracene-based sensors for CGM and the management of diabetes demonstrates the applicability and robust nature of these compounds. Yet only one of these ventures, SMSI, which became Senseonics in 2012, has been able to push beyond the initial stage of development and bring a long-term CGM, the Eversense CGM system, to the market for daily use by the diabetes patient community.

## Successful Example

After Czarnik’s departure from SMSI and changing their name, Senseonics continued their animal studies for safety and efficacy in rats, dogs, pigs, and monkeys. In each case, fully implanted sensors were evaluated for up to 6 months, exhibiting a first order kinetic decrease in fluorescent signal over time in response to glucose concentration.^41^

**Figure 6:**
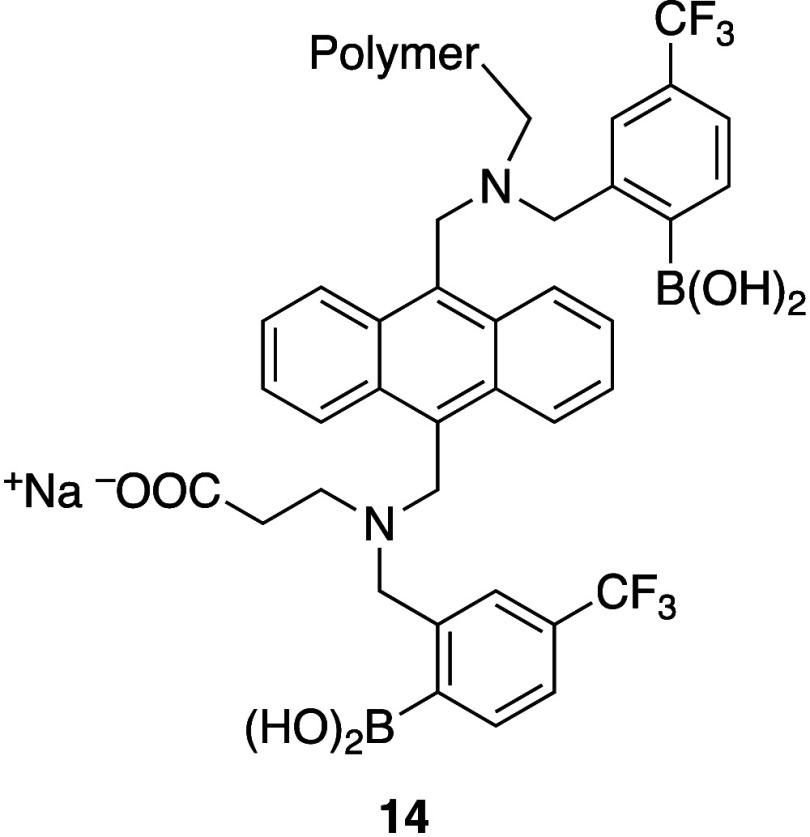
Diboronic acid sensor used in implanted CGM.

With these studies complete, human trials of the Senseonics CGM began. The small capsule shaped sensor (3.5 mm × 18.3 mm) containing a proprietary hydrogel sensor, based on the optimized diboronic acid **14**, grafted onto a PMMA surface was able to measure glucose concentration in ISF (Figure 6).^35^ The capsule itself was implanted in the subcutaneous space of the wrist by a health care professional (e.g., doctor, physician’s assistant, or nurse practitioner) under local anesthetic in a doctor’s office and included a fully functioning miniaturized microfluorimeter.^35^ This initial clinical version communicated wirelessly with an external reader (i.e., a watch worn by patients above the implanted sensor), which was responsible for powering the sensor and collecting, processing, and displaying the data.

Unfortunately, unlike in animals, sensors implanted in humans lost their signal during the first day after implantation.^35^ Analysis of failed sensors showed that the diboronic acid had undergone oxidative deborylation to produce phenols that were not sensitive to glucose concentration. Such oxidative deborylation likely occurred due to the patient’s initial inflammatory immune response to the implanted sensor. To the best of our knowledge, it remains unclear why a similar issue was never observed in animals.

In order to prevent oxidative deborylation in humans, two approaches were investigated: (1) platinum sputter coating of the porous hydrogel sensor^35^ and (2) incorporation of a silicone dexamethasone acetate “collar” around the sensor to attenuate the impact of the patient’s inflammatory response.^42^ In the first case, a 3 nm layer of platinum metal was sputter coated over the oxidatively sensitive hydrogel containing trifluoromethyl substituted sensor **14**. The platinum layer protected the boronic acids from oxidation by reactive oxygen species (ROS) at the implantation site and extended sensor stability *in vivo* from hours to at least 6 months. Originally, using the platinum nanolayer to stave off biological fouling was preferred, as it provided a simple and uncomplicated safety profile. However, upon application to the clinic, it was determined that slow release of dexamethasone acetate into the patient by the collar, a method used in other medical devices to decrease inflammatory response, was well tolerated by patients; clinical trials found no dexamethasone in the blood of patients (collected through regular blood draws) who had implanted sensors equipped with a dexamethasone acetate collar.^42^ As such, the incorporation of a dexamethasone collar on each Senseonics CGM sensor has been the standard of practice over a number of generations of the device.^43^

The first fully successful clinical trial of the Senseonics CGM was reported in 2014.^44^ In this study, patients utilized the implanted CGM for 28 days—two times as long as any other CGM currently on the market. The fully implanted sensor exhibited greater accuracy, compared to other commercially available enzyme-based CGMs that are inserted transdermally by patients (e.g., FreeStyle Navigator CGM, DexCom SEVEN CGM, and Medtronic CGM),^33^ as determined by mean absolute relative difference (MARD) between paired SMBG reference measurements (i.e., finger prick) and CGM measurements. Further, the stability of the boronic-acid-based sensor over 28 days suggested that a much longer lifetime should be possible without loss of accuracy.

**Figure 7:**
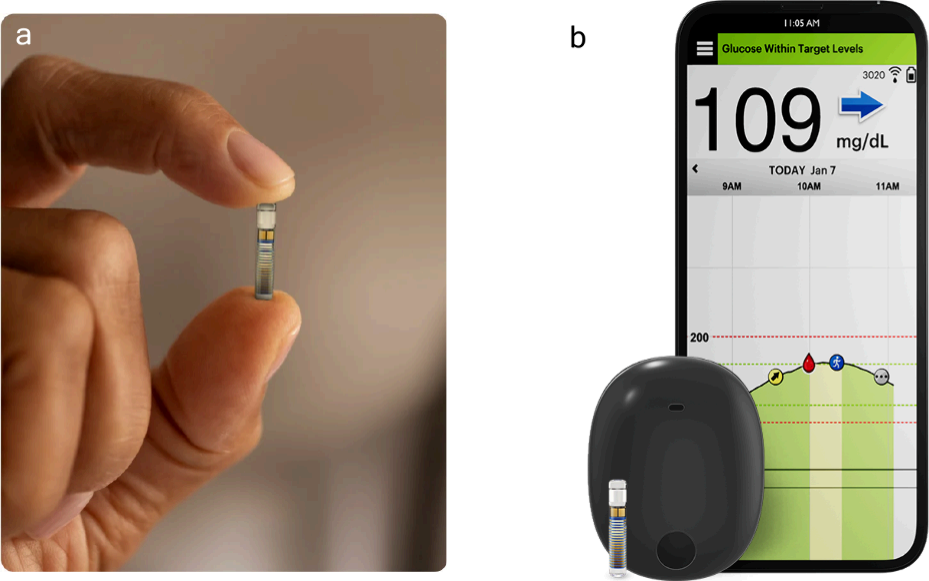
(a) Senseonics Eversense miniaturized microfluorimeter implanted in subcutaneous tissue of the upper arm, (b) sensor, removable external transmitter which adheres to the skin of the upper arm, and smart phone application.^7^

Further clinical trials iterated on this original CGM, which was now referred to as the Eversense CGM.^45,46^ 90-day studies in humans showed that this CGM was safe and effective,^43,47^ leading to it gaining the CE mark in Europe and South Africa in 2016 and subsequent FDA approval for its use in the U.S. in 2018. In these studies, the small sensor was implanted into the subcutaneous space in the upper arm rather than the wrist, providing significant improvements in patient comfort and allowing for the removable receiver/power supply to be worn on the arm (Figure 7). Further, patients could now interact with a smartphone application, rather than a watch. Real-life data from the first cohort of commercial users of the Eversense System in the U.S. showed that the fully implanted CGM provided excellent performance under real-world conditions throughout the 90 days of the trial.^48^

Following on these positive results, the PRECISE^49^ and PROMISE^50^ studies were launched to evaluate the Eversense CGM system over a 180-day (6-month) duration. Containing an updated algorithm, based on the data gathered during the previous 90-day trials, this version provided the most accurate blood glucose levels based on ISF fluorescence measurements to date.^43,47,48^ Similar to prior trials, good safety and efficacy profiles were observed, and only a small number of sensors needed to be replaced prior to the full 180 days, as indicated by an alert provided by the sensor in conjunction with the smartphone application. The Eversense E3 180-day sensor received both its CE mark in Europe and FDA approval in 2022.^51^ As of this writing, a new 365-day iteration of this CGM has been approved by the FDA for use by U.S. consumers.^52^

Having shown its value to patients, the Eversense System became the first long-term CGM to receive FDA approval as an integrated continuous glucose monitoring system (iCGM) through the FDA’s De Novo pathway in April 2024.^53^ iCGM systems are integrated with an insulin pump, allowing algorithms to predict and respond to hypo- and hyperglycemic events and trends without patient input. While the currently approved system is still considered to be hybrid (i.e., patients still manually provide a bolus of insulin near meals), the integration of CGM with an automated insulin pump has been shown to increase the time a patient spends in their target glucose range and to improve overall disease management.^54^ While the long-term goal remains to develop a fully automated closed-loop long-term artificial pancreas system that will anticipate and respond to blood glucose levels without any patient intervention, Eversense iCGM moves the diabetes community one step closer to this ideal.

## Conclusion

After more than 30 years of research and development, the simple boronic acid fluorescent chemosensors **3**, **4**, and **5** that we disclosed in the 1990s have developed into a state-of-the-art long-term (i.e., 365-day wear) CGM that can be integrated with an insulin pump. As we look ahead, we anticipate a fully automated artificial pancreas system will be possible using this platform.

Reflecting on our simple ponderings about whether reversible boronate ester formation could allow for the fluorescent sensing of neutral saccharides, we are gratified to have been part of fluorescent chemosensing from the beginning. We hope that others are encouraged by our story and continue to convert basic observations into technologies that can improve the lives of real people.

## Supporting Information Available

Web link for CGM sensor implant experience (PDF)
