# Supplementary material for: Fluorescent Chemosensors in the Creation of a Commercially Available Continuous Glucose Monitor
Source: ACS Sens. 2024 Nov 25;9(12):6320–6. doi: 10.1021/acssensors.4c02403 (PMC11686512; doi:10.1021/acssensors.4c02403)
Supplement: Supplementary file 1 — se4c02403_si_001.pdf [file se4c02403_si_001.pdf]

This video recounts one author's (AWC) patient experience with the implantation process for the Senseonics Eversense CGM. The mp4 file, which is too large for upload, can be found at the link below.

https://drive.google.com/file/d/1vXh3SUjTHT5NbD4exInhrQ344V1zY4In/view?usp=sharing
